# Double trouble: COVID-19 vaccine misinformation amidst conflict in Ukraine

**DOI:** 10.1016/j.amsu.2022.104127

**Published:** 2022-07-09

**Authors:** Tharwani Zoaib Habib, Farah Ennab, Lolita Matiashova, Faisal A. Nawaz, Anastasiia Volkova, Vira Trill, Mohammad Yasir Essar

**Affiliations:** Faculty of Medicine, Dow Medical College, Dow University of Health Sciences, Karachi, Pakistan; College of Medicine, Mohammed Bin Rashid University of Medicine and Health Sciences, Dubai, United Arab Emirates; LT Malaya Therapy National Institute, National Academy of Medical Sciences of Ukraine, Kharkiv, 61039, Ukraine; College of Medicine, Mohammed Bin Rashid University of Medicine and Health Sciences, Dubai, United Arab Emirates; Lviv National Medical University, Lviv, Ukraine; Kabul University of Medical Sciences, Kabul, Afghanistan

## 1 Introduction

The repercussions of the COVID-19 pandemic have presented valuable insights into new public health challenges which were once difficult to recognize. At the core of this strenuous public health emergency, lies a dire urgency to prioritize effective therapeutic interventions - namely vaccines, surveillance programs, and public awareness measures. Particularly in the midst of a digital era fueled by misinformation and ongoing conflicts [1]. On the 24th of February earlier this year, Ukraine was embattled by recurrent conflicts with Russia, its neighboring country; exacerbating the number of people potentially affected by this humanitarian crisis and causing profound destruction to medical institutions which encompass the cornerstone of treatment for many patients affected with COVID-19 [2]. Additionally, there have been increasing concerns of a significant decline in COVID-19 testing since the beginning of this conflict, suggesting that undetected transmission is occurring at a much higher rate than previously reported [3].

Vaccinations have been proven to be the most effective intervention in battling the COVID-19 pandemic, yet this primary tool of prevention hasn't been well utilized or sufficiently promoted by the Ukrainian government [4]. As the COVID-19 vaccine campaigns in Ukraine started one year before the ongoing conflict with only around 36.93% of its population receiving two doses of the vaccine, this strikingly low level of vaccination deemed Ukraine as the least vaccinated country in Europe [5]. Along with the ongoing conflict, the rate of vaccination doesn't appear to be on the rise anytime soon. The justifications behind this anti-vaccine movement in Ukraine vary, but the most notable one which appears to have strong implications amongst the public is the growing wave of vaccine “misinfodemic”. This emerging wave of vaccine misinformation during the ongoing conflict has the potential to displace the public's trust and result in a significant resurgence of COVID-19 infections [6].

In view of this alarming humanitarian catastrophe in Ukraine, bound by numerous anti-vaccination campaigns, there are prudent grounds for concern regarding vaccine confidence and public uptake [7]. Combating misinformation is an essential factor in cultivating an environment where infectious diseases spread less rapidly, both in terms of cases and in the public's perception. The aim of our commentary is to highlight the implications of this emerging “misinfodemic” on vaccine acceptance during a critical situation and suggest key recommendations for enhanced community-oriented public health measures.

## 2 Ongoing vaccine “infodemic” on social media platforms

Even though the vaccination campaigns continue in Ukraine, their progress has fallen rapidly and has completely stopped in the temporarily occupied territories and the conflict zones. Since the outbreak of hostilities, no new strains of covid-19 have been detected, and testing for covid itself has fallen by 90% [8]. The Ministry of Health of Ukraine last announced official statistics on COVID-19 on February 24, 2022 [9]. Although vaccination is actively continuing in Ukraine, its level has dropped significantly, and in two regions, it has completely stopped due to occupation [10]. To make matters worse, most vaccines expire in July and August 2022 and have targeted 70% of the population. It is also a rather big challenge that due to low diagnostics people are less aware of the real situation caused by COVID-19 and tend to seek medical care less frequently now [11,12]. Additionally, in many regions of Ukraine active shelling continues, limiting the ability to consult a doctor in person.

Long before the start of the military conflict in Ukraine, misinformation had spread widely enough to interfere with the vaccination campaigns, and in the present conditions, it has rapidly worsened [13]. Ukraine has started its vaccination campaigns much later compared to other countries, potentially giving ample time for misinformation to spread on social media snatching information out of context at the expense of the vaccines' side effects. The most commonly circulated pseudo theory about the connection between 5G and COVID-19 was the presence of a chip in vaccines and the artificial origin of the virus, furthermore, anti-vaccination activists have been vocal in their opposition and theories going on strikes to get more people's attention [14].

Despite the current misinformation in Ukraine, a campaign was recently launched by the Ministry of Health to fight against misinformation, posting various information on social networks. Unfortunately, the voice of misinformation turned out to be louder as 42% of Ukrainians continue to receive their information from social networks from unreliable sources [15]. Thus, despite the authorities' efforts, the misinformation waves continue growing and developing. The military conflict in Ukraine brought not only a humanitarian catastrophe but also an infectious one; providing favorable conditions for the dissemination of false information**.**

## 3 Potential implications of vaccine misinformation

Anti-vaccination campaigns in Ukraine started around 15 years ago, hence, vaccine hesitancy is not a new concept to Ukrainians. This is shown by the fact that only 53% of children were vaccinated for polio in Ukraine as this is also the case with measles and diphtheria vaccination rates [16]. These anti-vaccination campaigns could create anxiety and fear among the 42% of Ukrainians who are willing to get vaccinated for COVID-19 and who might end up not getting vaccinated due to the misinformation and rumors [17]. In addition, these campaigns will also prove to add up to the reason for the other 56% of people who were not willing to get vaccinated in the first place.

Since the war began in Ukraine, at least 31 attacks have been reported on healthcare in which 24 healthcare facilities were destroyed, leading to 12 casualties and 34 injuries [18]. These attacks meant limited accessibility for the citizens to healthcare facilities in the country and if low COVID-19 vaccination rates persist, patients will not be able to get treated, which will lead to increased COVID-19-related deaths. Moreover, after the Russian military intervention in February 2022, the predicted COVID-19 cases went up to 30,000 cases daily [19]. Following this prediction, it got nearly impossible to follow up on the actual case count due to limited testing facilities, especially in hostile territories. This is shown by the Worldometer dashboard which reported 5000 cases per day in Ukraine which is six times less than the expected count. This is due to the undocumented spread of COVID-19 which exposes hundreds and thousands, maybe even millions, of citizens to the risk of contracting COVID-19 which can potentially lead to several deaths in Ukraine. The only way to counter this spread is through vaccination, which is, unfortunately, being hindered by the spread of vaccine misinformation in the country [20].

The ongoing war in Ukraine not only directly affected the healthcare system but has also affected it indirectly with Ukraine's economy suffering a huge loss due to the Russian invasion which led to limited supplies especially oxygen availability for the healthcare system [21]. Along with the lack of humanitarian assistance, this means that Ukraine is not ready to face any future waves of COVID-19 due to new variants coupled with emerging waves of vaccine misinformation and rumors.

Following the recent improvements in controlling the COVID-19 spread, remittances to Ukraine have been expected to rise by 20% in 2022, which can help improve the infrastructure of the country and fulfill the basic necessities of the population. However, if low COVID-19 vaccination rates persist due to spreading misinformation, new variants of COVID are likely to develop, which will again redirect these funds to be used in improving healthcare facilities in order to deal with the burden of new COVID-19 cases, limiting the recovery of the country from war and hindering any future developments [22].

COVID-19 not only has pronounced effects on the extremes of ages, i.e. elderly and children but also affects pregnant women and their unborn children. It has been reported that COVID-19 can lead to an increased incidence of pre-term births, C-section delivery, low birth weight, pre-eclampsia, and various other pregnancy-related complications [23]. This would lead to the prolonged stay of the mother and their child in the hospital burdening the already suffering system. Therefore, it is essential to counter the spread of misconceptions and fake narratives regarding COVID-19 vaccination in order to prevent any future waves and COVID-related deaths.

## 4 Recommendations for change

Several actions can be pursued in order to counter vaccine misinformation in Ukraine and ultimately increase COVID-19 vaccination rates. One of the important steps involves the role of social media in disseminating authentic medical information related to COVID-19 and its implications and highlighting the importance of COVID-19 vaccination. The significance of social media in COVID-19 vaccine promotion was shown by a cross-sectional study which stated that 37% of their participants reported that social media had a positive impact on their willingness to get the COVID-19 vaccine [24]. This is further elaborated by a German study that expands on the importance of the language and communication barrier in the limited COVID-19 vaccination rates among migrants. This study hypothesizes that addressing the language barrier through social media can potentially increase the vaccination rates among migrants by up to 14%. Thus, these facts prove that social media can have a vast influence over the majority of Ukrainians in order to increase COVID-19 vaccine acceptance [25].

Another important step that could be implemented is coordinating with countrywide community educational programs which can target other populations including school children and vulnerable populations such as pregnant women and the elderly. These programs can be valuable in the sense that these groups are not very familiar with social media, especially the elderly and the children, therefore, they can benefit from these programs and help spread accurate information to their families, ultimately increasing the COVID-19 vaccine acceptance. The importance of this action is presented by an Israeli cross-sectional study which shows that over 78% of the participants who were vaccinated for COVID-19 had a higher level of overall knowledge about COVID-19 [26]. This means that the success of these community-based educational programs could have a major influence on the rates of COVID-19 vaccination in Ukraine by eliminating the misinformation and developing trust among the population.

In order to prevent the vaccine misinformation in Ukraine it is first necessary to measure the extent of it, so that an accurate plan can be developed to counter it. To do so, the government should introduce surveillance programs in the cities as well as in the rural areas so proper actions like education programs and advertisements can be implemented in different parts of the country in order to spread awareness and thus increase the COVID-19 vaccination rates throughout the country. Not only should the government introduce surveillance programs, they should also identify the sources spreading misinformation and take immediate actions of shutting these down if they fail to comply with national guidelines.

The emerging use of Artificial Intelligence (AI) in the modern world can prove to be helpful in countering the COVID-19 “infodemic” of rumors on social media and other online platforms. A similar plausible action is being taken by researchers at the Stevens Institute of Technology who are working on developing such facilities which can cross-check online articles against reputable and trusted media websites and remove any articles which contain any incorrect information regarding COVID-19 and its vaccines [27]. Currently, the team's algorithm is calculated to have an 88% accuracy rate which means that if further work is done in this field, especially in Ukraine, misinformation can be significantly lowered which will prove to be beneficial for the medical society and can potentially increase the COVID-19 vaccination rates in the country.

Lastly, arranging broadcast sessions with medical professionals can help by spreading awareness regarding the benefits of COVID-19 vaccines and demonstrating the minimal side effects that they may cause. The importance of this action is proved by a cross-sectional study performed in Pakistan which showed that 44% of their participants agreed to get vaccinated for COVID-19 if recommended by a physician [28].

## 5 Conclusion

As the challenge of vaccine hesitancy and misinformation related to COVID-19 continues to threaten health systems worldwide, Ukraine has also been struggling with a conflict-driven catastrophe. Misinformation is causing an indirect toll on the physical and mental well-being of Ukrainians, which further adds to the impending health and socio-political crisis in the country. Further research is warranted to understand the impact of misinformation on public health in order to devise early interventions and national policies for building vaccine confidence in the Ukrainian community.

## Sources of funding for your research

None.

## Ethical approval

N/A.

## Consent

N/A.

## Author contribution

All individuals who meet authorship criteria are listed as co-authors who have participated adequately in this work to take public responsibility for the generated results and content, including participation in the idea, design, writing of the manuscript, or revision. Furthermore, each author listed certifies that this material or similar material has not been and will not be submitted to or published in any other publication before its appearance in **The Annals of Medicine and Surgery.**

Conceptualization: FE, FN, ME; Writing original draft: ZT, FN, LM, GN, FN, KN, AV, VT, ME; Review and editing: FE, FN, AV, ME.

## Registration of research studies

1. Name of the registry: N/A.
2. Unique Identifying number or registration ID: N/A.
3. Hyperlink to your specific registration (must be publicly accessible and will be checked): N/A

## Guarantor

N/A.

## Declaration of competing interest

None declared.
